# Socio-cultural contexts of end- of- life conversations and decisions: bereaved family cancer caregivers’ retrospective co-constructions

**DOI:** 10.1186/s12904-017-0222-z

**Published:** 2017-08-14

**Authors:** Jennifer Nyawira Githaiga, Leslie Swartz

**Affiliations:** Department of Psychology, Stellenbosch University, Private Bag X1, Matieland, Stellenbosch, 7602 South Africa

**Keywords:** End-of-life, Communication, Palliative care, Family caregivers, Focus groups

## Abstract

**Background:**

End-of-life communication becomes increasingly difficult in terminal cancer, which inevitably entails conversations around dying and death. In resource-limited areas, the context of end-of-life communication is usually home-based palliative care comprising mostly women in the family who play critical roles as informal caregivers. This article examined the content and contexts of family end-of-life conversations and decisions based on the retrospective accounts of a sample of bereaved women family cancer caregivers in Nairobi, Kenya.

**Method:**

An interpretative phenomenological analysis approach was utilized to explore pertinent end-of-life communication themes. Four mini focus group interviews with a total of 13 participants [*n* = 5; *n* = 3; *n* = 3; *n* = 2] were conducted.

**Results:**

Two end-of-life themes, advance directives as preparedness for death, and initiating death talk were examined. Findings (a) illustrate the role of family dynamics in influencing the nature of end-of-life conversations and decisions (b) demonstrate the transitional nature of family caregiver roles, and (c) underscore the paradox of the critical role played by family members in palliative care versus their ill preparedness in dealing with end-of-life issues.

**Conclusions:**

Findings are relevant in informing palliative psychosocial interventions and specifically the concerns and decisions of cancer patients and their families. This prompts further engagement with the question of how to equip family caregivers in resource-limited contexts for end of life care. Methodologically, these results demonstrate the possibility of simultaneous elucidation of individual experiences, interactive co-constructions and the socio-cultural contexts of experiences and meaning making processes in IPA research.

## Background

End-of-life communication is a well-researched phenomenon amongst terminally ill patients, often in liaison with healthcare professionals [1, 2]. Studies show that communication becomes increasingly difficult in terminal cancer, which inevitably entails conversations around dying and death [3–5]. The cited studies illustrate that deterrents to such communication include fear of stirring up emotional distress, attempts at mutual protection by patients, family members and health professionals, and the quest to keep hope alive through engaging in positive conversations. In addition, pre-existing patterns of family communication prior to the onset of illness, which avoid difficult or distressing subjects, are also likely to deter frank discussion at this stage [6, 7].

An implicit assumption in much of the palliative care literature is that palliative care takes place in the context of a reasonably well resourced health system, where there are sufficient resources (institutions and trained personnel) to cater for the needs of patients along the various trajectories of illness. A contrasting scenario is evident in palliative care in Sub-Saharan Africa, characterized by under resourced healthcare systems. In this context, pre-dominant palliative care models include hospital care, community home-based care and, integrated community and home-based care [8]. Both models, initially established as a response to the HIV/AIDS pandemic, target service provision in areas with limited access to public healthcare facilities many of which are in rural towns [8–10]. Consequently, some urban dwellers may not have access to these services.

Alternative models of palliative care in urban Africa include home-based care with supplementary services of paid nursing care, funded either privately by families or through medical aid organizations, as well as inpatient care in nursing homes, frail care facilities, hospices and hospitals, as illustrated in two South African based studies [11, 12]. Access to alternative palliative care is associated with affluence, which determines who can afford to pay for such services and/or who has access to health insurance that can cater for these services.

In Kenya, where the current study is based, the concentration of specialized cancer management services and oncologists in Nairobi [13, 14] has led to movement of cancer patients from rural areas to Nairobi to seek specialized services, most of which are palliative in nature. This bears implications for end-of-life care, which is often delegated to family members and relatives residing in Nairobi. As illustrated in this article, caring for patients with incurable cancer often occurs at home, with a family member taking primary responsibility for care, and hospitalization when patients are deemed too ill for informal home care. In addition to being a hub of cancer care, cosmopolitan Nairobi is significant as a melting pot of cultures. This shapes end-of-life discourses as terminally ill patients and their families simultaneously straddle cultural community beliefs and more contemporary beliefs about illness and dying. Against this background, there is a need to better understand the experiences of families of terminally ill cancer patients in an urban context (Nairobi) with a view to contributing to research on culturally relevant end-of-life care [15].

This article seeks to contribute to this need by examining the content and contexts of family end-of-life conversations and decisions based on the retrospective accounts of a sample of bereaved women family cancer caregivers in Nairobi. The following questions are addressed: What was the nature of family interactions? Who initiated end-of-life discussions? How did family members, caregivers and cancer patients respond to such conversations? Which cultural discourses featured in these conversations? This paper is developed from a broader study which explored the lived experiences of family cancer caregivers in Nairobi [16].

## Method

### Participants

Sources from a local hospice, personal contacts and referrals from participants (snowball sampling) were employed to gain access to bereaved women family cancer caregivers living and working in Nairobi. Thirteen caregivers participated in the study. Though men also serve as caregivers, this study specifically focused on women because, in this context, caregiving in Africa is a gendered role ascribed to women who often comprise the majority of informal family carers [17–19].

### Procedure

Ethics clearance to conduct the doctoral study from which this article is developed was obtained from University of Cape Town Research Ethics Committee, Department of Psychology [16]. This ethics approval granted clearance to approach various gatekeepers for assistance in sourcing participants. At the time no further ethics approval from Kenya was required as this was prior to the establishment of Kenya’s National Commission for Science, Technology and Innovation (NACOSTI) Act 28 of 2013 which, as of 2014, currently requires all persons conducting research in Kenya to obtain a research permit in addition to institutional ethics approval. The study did not include cancer patients and, thus, no approval from the medical health departments in South Africa or Kenya was required. Participation was voluntary. During initial consultations with potential participants, JNG discussed the nature of the study and gave opportunity for questions and clarifications. This entailed clarifying that study findings would be utilized for academic research purposes and that participants’ identities would be safeguarded in the process. Prior to the start of a focus group discussion, each participant gave written informed consent including permission to audio record the focus group discussion. Further, each group member gave verbal consent for a research assistant to sit in and take down observational notes.

Focus groups were conducted by JNG with the aid of two research assistants, one who assisted with logistics of setting up for focus groups and another who wrote down observational notes during focus groups. The focus groups venues, all of which were in Nairobi and easily accessible to all participants, were determined in consultation with participants. In order to allow both for individual views and for issues to be discussed and co-constructed, four mini focus group discussions (FGDs) [20, 21] were held. A detailed explanation of the rationale for the use of these focus groups is furnished in a methodological article [22]. The small group format was especially appropriate to this context; in many African contexts issues of health and care are negotiated and discussed in community discursive contexts. In addition, a number of participants requested to be interviewed in small group format. The first focus group lasted over 3 h with participants deeply engaged in conversations about their experiences. The researcher thus decided to reduce the group sizes of subsequent focus groups to give ample time for sharing of individual narratives as well as group conversations around these accounts. The average time for each subsequent focus group was 2 h. Discussions were held in English.

### Data analysis

The study utilized an Interpretative Phenomenological Analysis (IPA) approach, which focuses on in-depth investigation of peoples’ significant life experiences, meanings ascribed to such experiences and further, how individuals make sense of these experiences [23]. IPA procedures for data analysis [24], which are adaptable for focus group data analysis, served as guidelines [25, 26]. Data analysis, in which focus groups were analysed consecutively, involved several readings of each focus group transcripts while writing down observations and comments (exploratory, linguistic and conceptual) on a margin alongside the numbered meaning units in each transcript [24].

The interpretation process entailed a moving back and forth between individuals’ subjective experiences as well as shared aspects of experiences collaboratively negotiated in the course of focus group exchanges [25, 26]. In addition, hermeneutic process involved: real-time sense-making taking into account the chronological order in which participants narrated their experiences, and; the deconstruction of events and organization of these into thematic ideas, that is, post-hoc meaning making [26]. Emergent themes based on experiential claims were listed and further clustered into broader themes. Following analysis of each focus group, a thematic analysis across the 4 groups, foregrounding points of convergence and divergence, was undertaken. The following section explores two end-of-life themes that arose in FGDs.

## Results

In this section, we present some findings from the four FGDs. FGD 1 comprised 5 women aged 34–52 years, all professionals working Nairobi, who had cared for a family member with terminal cancer. FGD 2 comprised 3 elderly widows (65–75 years) each of whom cared for a spouse with terminal cancer. FGD 3 comprised 3 women aged 31–36 years who each cared for a terminally ill parent, while FGD 4 consisted of 2 women aged 27 and 33 years who served as caregivers of siblings with terminal cancer.

Utilizing relevant illustrations from FGDs, this section discusses the content and contexts of family end-of-life conversations and meanings ascribed to these conversations. Two related themes are presented: (1) advance directives as preparedness for death, and (2) initiating death talk. These themes were developed from JNG’s dissertation chapter on death, dying and bereavement transitions in the context of family caregivers’ experiences of ‘midwifing death’ [27]; watching and waiting on their terminally ill loved ones as death approached. We utilize the term advance directives in the broader sense of and verbal or written instructions conveying ‘a person’s health care preference while they are competent to make decisions for themselves….’ ([28], p.1) In our specific context advance directives include wills, do-not-resuscitate (DNR) directives, and other variations of end-of-life conversations and decisions regarding terminally ill cancer patients, and from the perspectives of lay family caregivers. The symbol [] indicates omitted sections of text. Pseudonyms are utilized to safeguard participants’ identities.

### Advance directives as preparedness for death – ‘it’s not that I’m dying right now’

The theme of wills generated substantial discussion among focus group participants. Wills were considered a hallmark for preparedness or lack of preparedness for death as illustrated in the following excerpts drawn from an FGD 1 conversation:

**Elimu (FGD1)**: Before he [husband] went to the hospital we sat with the family and he was telling us ‘now very soon you’re going to have one parent and it’s good you start adjusting. Mum cannot afford the rent. Now, she’s going to look for a smaller house [] And the little money which I’m going to leave will educate the ones who are in school’ [] And he said ‘Jumuia[daughter] take that book and write what I’m saying.’ [] And then after that he was saying ‘it’s not that I’m dying now, right now [] But you know my sickness; you know it’s cancer and there’s no cure’ [] I wouldn’t have managed but he really prepared us very well.

The statement ‘it’s not that I’m dying now’ points to the commonly held Kenyan belief that speaking about death implies summoning it, which might explain Elimu’s husband’s need to reassure his family that he was not on his death bed at the time of issuing his will. Retrospectively, Elimu appreciated her husband’s advance directives in aiding her cope with his death.

Elimu’s experience seems to have caused other group members to recall and reflect on their own experiences, with Elimu’s story as the point of reference for Baraka and Usafi’s meaning-making:

**Moderator:** I don’t know if anyone has something to say as we part. What would you say to other women who are going through this as we end our discussion?

**Baraka (FGD1)**: Well, the only thing I’d say, I’m very happy that your [Elimu’s] husband did that because my mother didn’t do that and it left us in disarray [] I think that’s very helpful because reality is reality whether you like it or not [] and I kept on telling my sisters this and actually they thought I was an enemy. At one point they just told me ‘you want your mother to die’ which is not the case [] I didn’t know the date but from my senses I could tell that this is not the road to Jahazi Town [parents’ current residence]. This is the road to Mpakani Town [rural home] where the grave was going to be put [] and this is where I *really* feel as a family we went wrong and we did not support my mum because we were not giving her strength to, to, to be *dignified* [] Just be like this gentleman who died because I think it was a very dignified death.

Baraka sought to initiate end-of-life discussions with her sisters in an effort to support her mother by facilitating what she deemed a dignified death. This yielded dissonance between cultural beliefs regarding speaking about death being perceived as a death wish versus the reality of impending death, communicated in Baraka’s metaphor of ‘the roads’, symbolizing the common Kenyan practice of burial in rural homes. In this cultural context a death wish may be interpreted as ill will towards a seriously ill family member in the sense that ‘you want your mother to die’ in contrast to waiting for the ill person to communicate one’s dying wishes, like in Elimu’s case. However, Baraka’s intent seemed to be encouraging ‘death talk’ in the context of preparation for death, a perspective documented in palliative care literature [1, 2, 29]. The context of the cited studies – established palliative care facilities with palliative care professionals - differs from the current study where end-of-life care conversations occurred in home or hospital contexts outside of formal palliative care structures.

Usafi picked up the conversation, incorporating both Elimu and Baraka’s experiences in her meaning making:

**Usafi (FGD1)**: Yes, I just wanted to identify with eh, Auntie [Elimu]. For me, I think what helped us as a family is being prepared. Dad has been sick for a long time but it wasn’t until last year June that he sat us [family] down and told us ‘I want you people to walk with us this journey’. I remember sharing with a friend and I’m telling her ‘hĩĩ! The way this man has shared, it’s like he’s going to die the next minute’[] so he wrote his life story – he actually gave it to us [] and so we safeguarded the family history. He told us where he wants to be buried [] when I heard you [Baraka] talk, I was thinking your mum hid so much. She didn’t want to see you in pain and sometimes for a woman it’s harder. For a man I think he’s feeling like ‘if I leave my family like this everything will be, you know, in disarray’.

Similar to Elimu’s account, Usafi and her family were invited to play audience as her father gave advance directives. For Usafi, this entailed navigating the tension between the traditional belief that speaking about death is summoning it and respecting her dying father’s wishes to discuss his end-of-life concerns with the family.

Usafi’s last comment supports gendered socialization of (a) men as the heads of patrilineal households with responsibility for decision-making in the home [30], and (b) women as carers and nurturers of their children and spouses, including caring for ill family members [31, 32]. Hence, it is understandable how women’s concern at the end of life would be to ensure the emotional comfort of their families which might include avoiding ‘death talk’ to avoid upsetting them. This resonates with the sentiments of a sample of South African cervical cancer patients some of whom were concerned about the potential emotional distress to their families upon disclosure of their diagnosis [33]. However, the following section demonstrates that beyond gender, there were other factors in play.

### Initiating ‘death talk’: Whose call? – ‘he wouldn’t talk’

Huruma, an elderly widow in FGD 2, found herself caught in the tension between her children’s demand for their father’s will and her husband’s silence about it:

**Huruma (FGD2)**: I actually remember telling my husband to write a will or to discuss with me what he wants with his, with the properties [] because my son kept on telling me ‘mum, you people have to write a will.’ And I kept on talking to my husband [silence, then lowers voice] and his mouth was tight-lipped. He wouldn’t; he wouldn’t say [] he wouldn’t say a single thing [brief silence] and in fact I, I felt like I was pressurizing him because my children were also telling me ‘*talk to him, ask him* [spoken in whispers but emphatic] has he got a will?’ but he wouldn’t talk [silence] he would not say a thing.

**Jahazi (FGD2)**: He did not believe in wills

**Huruma (FGD2)**: No. He wouldn’t talk *at all*; he wouldn’t say. And I realized now I am pushing him or I am making him say things he doesn’t want to say because he would just keep quiet and look at me.

In addition to her primary caregiver role, Huruma was a mediator between her children and husband. One could sense her predicament as she was caught between her husband’s avoidance, the demands of her children and her quest to maintain family harmony. Jahazi’s comment ‘he did not believe in wills’ insinuates that refusal to issue his dying wishes may be linked to known cultural traditions. Huruma also alluded to the possibility that her husband may have considered being pressured to give his will inappropriate and thus resisted her attempts. Perhaps silence was his way of protecting his family from distress in light of his impending death as observed among terminally ill parents in an American based study that investigated reasons for avoidance amongst families coping with lung cancer [7]. Nevertheless, Huruma’s husband’s avoidance seems to negate Usafi’s idea of gender role socialization as a determinant of who should issue advance directives.

In another focus group Ojima’s 29 year old terminally ill sister was both explicit about her dying wishes and concerned for her family’s emotional wellbeing:

**Ojima (FDG4)**: She called my brothers and myself, sat us down, told us ‘you guys, this thing is terminal and you know, I’ll be gone pretty soon so you guys need to start dealing with it. Have you thought about therapy? I understand that the hospice, there’re people who can help you deal with, deal with this’. I was like ‘too much!’ You know we’re like ‘why are you saying that?’ My brother was like ‘stop talking; stop talking like that!’ She said ‘Seth, it’s terminal. The sooner you guys get to grips with it the better.’ [] She told my parents ‘you guys need to release me. I’m ready to go.’ [] she said all her goodbyes [] she ran us through her funeral program as she wanted it.

Ojima’s sister seemed intent on preparing her family for her death despite their unpreparedness evident in the siblings’ attempts to stop her from ‘talking like that’. Ojima’s sister’s recommendation for therapy suggests concern for her family’s emotional wellbeing and relational harmony, a theme featured in end-of-life discourses [34].

In certain instances, death talk featured directives regarding prolonging of life in terminal cancer. In the absence of explicit dying wills, caregivers and their families took responsibility for end-of-life decisions when their ill loved ones’ deaths were at hand [34]. Jahazi and Fikira (FGD2) recalled making difficult decisions close to their spouses’ deaths:

**Jahazi (FGD2)**: We were told that the doctor wants to see the immediate family[] he tried to explain to us that people may sometimes require that the patient be taken to ICU [intensive care unit] when the heart stops but he said he would, perhaps feel that that would be an unwise thing to do because of the bills that would be involved in ICU and yet you know it is a terminal illness [] But he cannot force any decisions on the family and the family would have to decide[] so we were asked []‘should the heart stop what would you like us to do?’ That was the hardest question and nobody was ready to answer it so we all kept quiet. We looked at him until I realized until I said something nobody would. So I said ‘we’ll go by his [the doctor’s] word and then we went round - every member of the family said ‘we’ll do what mum has said’ [] that was on Monday night. He passed away on [] Thursday night.

Rather than summon Jahazi, given that as the spouse and primary caregiver she would be considered the next of kin, he requested to meet the immediate family. The final decision was made jointly through family consensus.

Fikira’s contrasting experience reiterates the significance of family communal decision making in terminal illness:

**Fikira (FGD2)**: he [the doctor] called me and said ‘Mrs Fikira I want you to sign here. If your husband deteriorates at night I want you to give us consent that we don’t take him to ICU. If he was my father that’s what I would have done- he was a young doctor - because he is going to be on a life machine and this doesn’t help. But just tell me what you want.’ Now this is where the decision was *very* difficult [] I, I, signed and after the doctor had gone my daughter *cried*, sobbed, ‘mummy how can you *do* [emphasis] that? [] I wish he had told me before so that I consult the members of the family [] She was very bitter: ‘How can you do that? Sign our daddy out?’

In Fikira’s case, the doctor prevailed on her, as primary caregiver, to act as the sole surrogate decision maker [34, 35] on her husband’s behalf. Fikira’s response to her daughter’s outburst suggests the bone of contention was that the decision was made singlehandedly without consulting the other family members, who may have held different opinions.

Though it is unclear but possible that Fikira dealt with the same doctor, he involved Fikira’s family in the next major decision regarding their ailing father:

**Fikira (FGD2):** He [doctor] told us ‘tomorrow, Monday, come and see me with members of the family.’ We went on a Monday. He told us ‘we have done everything and now medically we’re not doing anything; it’s only nursing care. And there’re two things – you take him home or we take care of him in hospital: which one do you choose? Many people would like to see their person go [die] at home and even when they go home sometimes they get better.’ So we chose for him to come home [] on Wednesday he came home [] Friday he went - he died peacefully.

The decision regarding this advance directive, made in consultation with family members, did raise any objections. This discussion underscores the significance of cultural sensitivity, for example, in this context where family consensus was preferable to decision making by one family member [5, 34, 36].

Though both Fikira and Jahazi’s husbands were admitted in the same private hospital in Nairobi, Jahazi was neither offered the option of taking her husband home nor of being present with him at his death bed:

**Jahazi (FGD2):** I told the sister [nurse] I wanted to stay on for the night. And I knew they allowed people because there were other patients that were allowed to have a close member of the family with them when they became very sick, and she was adamant; she *refused* [emphatic] [] maybe she, they knew he was going and I felt *very* [emphatic] bad because they refused to allow me to, and then because they called me at 3.00a.m. [Silence] to tell me that he had passed on at 1.00a.m., could I go to the hospital and I said ‘no, I’ll come in the morning’. I mean, I couldn’t even drive.

Jahazi’s sense of disappointment in the healthcare personnel for denying her the opportunity to be present during her husband’s death and regret that she was absent at that critical moment is apparent. Possibly, her role as the informal primary caregiver was neither understood nor appreciated in the formal healthcare set up. Jahazi’s experience reiterates the sentiments of a sample of informal cancer caregivers who reported feeling invisible and side-lined by healthcare professionals [37].

This raises the question of why some families kept their relatives in hospital despite their knowledge that their illness was terminal; nine out of 13 patients died in hospital. This is consistent with a Botswana based study which showed more deaths, including cancer deaths, occurring in hospitals in urban cities [38]. Though the Botswana study did not elaborate on the reasons for hospital deaths, one possible explanation is that being ill prepared coupled with the lack of resources necessary for home based critical palliative care forced families to take their dying loved ones to hospital [15, 39]. This is in contrast to contexts where alternative resources such as home based and/or inpatient nursing and hospice care reduced the instances of hospital based deaths [11].

## Discussion

This article examined the content and contexts of family end-of-life conversations, decisions and meanings ascribed to such communication, through the lens of family caregivers’ experiences of midwifing the death of their ill loved ones. Two themes featured in focus group discussions amongst bereaved family caregivers: advance directives as indicators of preparedness for death (or lack thereof) and who initiates end-of-life conversations.

The study illustrates the role of family dynamics in influencing the nature of such conversations including when it is deemed the appropriate time for these conversations, who should or should not initiate these conversations, and who should be included in end-of-life discussions [3, 39]. Sometimes end-of-life conversations generated misunderstanding amongst patients and family members: (a) in home settings, when there were conflicts around emotional and psychological readiness for ‘death talk’ at a more pragmatic level versus cultural beliefs rendering such conversations taboo (b) in healthcare settings where there appeared to be inconsistencies in how healthcare personnel supported families of dying patients. The involvement of families underscores the relational nature of end-of-life discourses in this context. This may differ from cultural contexts at emphasize individual autonomy and agency, and where terminally ill patients take lead roles in end-of-life decisions, with support from palliative care professionals and sometimes family members [1, 2, 29].

Findings demonstrate the transitional nature of family caregiver roles. Whereas in their narratives several participants identified themselves as primary caregivers to their ill loved ones, caregiving emerged as a shared role particularly in the terminal phase where end-of-life decisions were negotiated at family level. Being a caregiver entailed straddling traditional beliefs, and more pragmatic perspectives about end-of-life conversations. In the absence of healthcare professionals assisting in home-based care, cancer patients, caregivers and their families struggled to navigate the unknown end-of-life terrain.

In certain instances caregivers were called upon to act as surrogate decision makers [34, 39], either as individuals or in liaison with other family members, within the formal healthcare system. The timing and haste with which such decisions were made placed an additional emotional burden on caregivers like Fikira who had to live with her decision not to take her husband into ICU despite her daughter’s outright disagreement with what she deemed ‘signing our daddy out’. Russ and Kaufman [35] posit that such end-of-life decisions are in reality not a matter of choice but rather one of endorsing the recommendations of healthcare professionals. The implication is that, though they often feel responsible, caregivers are not responsible for the outcomes of such decisions [37].

The main limitations of this study are methodological. First, the small sample size focused on idiographic understanding calls for caution in attempts to generalize the study findings. Second, data were based on retrospective accounts of bereaved family caregivers; hence some details may have been forgotten over time. Even so, some of the findings resonate with previously published studies providing a basis for comparative analyses end-of-life matters across varied contexts. For example, a Kenyan based study retrospectively examined advance directives of terminally ill patients based on hospital archived records in a private tertiary hospital [28]. Findings from this study show that 84.2% of the patients in this study who issued advance directives were cancer patients. Limitation of care documents and ‘do-not-resuscitate’ (DNR) were the most frequently reported types of advanced directives, while only 3 patients had living wills. The authors’ noted that the retrospective nature of their study was a limitation in exploration of various potentially mitigating factors including (a) patients’ knowledge and attitudes towards advance directives, and (b) the influence of patients’ relatives in decision-making around advance directives, both of which are addressed in this article. In addition, focus group interactions illustrate the value group synergy and collaborative meaning making in harnessing information that might have otherwise not been explored [40, 41]. Further, the findings demonstrate the possibility of simultaneous elucidation of individual experiences, interactive co-constructions and the socio-cultural contexts of experiences and meaning making processes in IPA research [26, 42, 43].

## Conclusions

This study shows the paradox of the critical role played by the family as ‘the central cultural and affective unit within which knowledge of terminal illness is processed and care for the dying performed’ [36, p.104] versus the ill preparedness of family caregivers (and families) in dealing with end-of-life issues [12, 39]. Findings bear implications for palliative cancer service provision in urban towns such as Nairobi, which is predominantly informal home based care with occasional hospital based care in critical phases. There is need to engage further with the question of equipping family caregivers with basic knowledge of end-of-life care. In addition, there seems to be potential for more liaisons between family caregivers and healthcare professionals involved in end-of-life care. Findings from this study are relevant in informing palliative psychosocial interventions and specifically the end-of-life concerns, needs and decisions of cancer patients and their families.

## Abbreviations

FGDs: Focus group discussions
ICU: Intensive care unit
IPA: Interpretative phenomenological analysis
